# Pumpkin and watermelon production constraints and management practices in Uganda

**DOI:** 10.1186/s43170-022-00101-x

**Published:** 2022-06-22

**Authors:** Fred B. Masika, Titus Alicai, Hussein Shimelis, Gabriel Ddamulira, Shahasi Y. Athman, Perpetua Ipulet, Morgan Andama, Arthur K. Tugume

**Affiliations:** 1 Department of Plant Sciences, Microbiology and Biotechnology, College of Natural Sciences, Makerere University, P.O. Box 7062, Kampala, Uganda; 2 Department of Biology, Faculty of Science, Muni University, P.O. Box 725, Arua, Uganda; 3 National Crops Resources Research Institute (NaCRRI), Namulonge, P.O. Box 7084, Kampala, Uganda; 4 School of Agricultural, Earth and Environmental Sciences, University of KwaZulu-Natal, Durban, South Africa

**Keywords:** Cucurbits, Uganda, Pumpkin, Watermelon, Production constraints, Food security, Nutritional security

## Abstract

**Background:**

Watermelons and pumpkins are cultivated in Uganda for their leaves, fruits, and seeds, thereby contributing to food, nutrition and income security. However, there is limited research and information on constraints affecting their production. This study assessed the current production constraints for watermelons and pumpkins, management practices, sources of production inputs to guide research and decision making in production of these crops.

**Methodology:**

Watermelon and pumpkin fields totalling 105 located in 28 districts from nine sub-regions of Uganda were surveyed. Purposive sampling was conducted based on the importance and availability of watermelon and pumpkin fields in the sub-regions using a questionnaire administered to farmers on different practices, management strategies, and current production constraints. Data were analysed to determine the relationship between the source of seed, sale of their produce, constraints, and control measures of biotic constraints in the different sub-regions.

**Results:**

Pumpkins and watermelons were grown by 85.7% and 14.3% of respondent farmers, respectively. The constraints as ranked by the farmers were pests, diseases, drought, high transport and labour costs. Bacterial wilt, downy mildews, anthracnose powdery mildews and virus diseases in this order were the most common and important disease constraints.

The whitefly (*Bemisia tabaci,* Gennadius), order hemiptera family aleyrodidae, aphids (*Myzus Persicae*, Sulzer), order hemiptera family aphidadae, melon fly (*Bactrocera cucurbitae,* Coquillett), order diptera family tephritidae and cutworm (*Agrotis ipsilon,* Hufnagel), order lepidoptera family noctuidae, were reported as the most limiting pests of watermelon and pumpkin production. Mixing of several agrochemicals was observed in watermelon fields coupled with gross lack of knowledge of proper usage or purpose of these chemicals may result in pesticide resistance, health and environmental hazards.

**Conclusion:**

Pests, diseases, and drought constitute the main constraints affecting watermelon and pumpkin cultivation in Uganda. Whereas weeding using hand hoes is the most common method of weed control, application of ash was the main strategy for pest management in pumpkin, while in watermelons, pheromone traps and pesticides were frequently used.

## Background

Pumpkin (*Cucurbita moschata,* Duchesne) and watermelon (*Citrullus lanatus,* (Thunb.) Matsum. and Nakai) belong to the genera *Cucurbita* and *Citrullus*, respectively, in the family cucurbitaceae, collectively referred to as cucurbits. They are cultivated in tropical and sub-tropical climates (McCreight, 2016; Rolnik & Olas, 2020). In developed countries, they are exclusively grown in monoculture systems (Lebeda et al., 2005). In developing countries, they are mostly cultivated in small traditional gardens (gardens found at the backyard of most homesteads) with low or no external inputs like fertilizers, pesticides, herbicides, and chemicals for disease control (Lebeda et al., 2005). The total global production of watermelons, pumpkins, squash (*Cucurbita maxima,* Duschesne), and gourds (*Lagenaria siceraria,* (Molina) Standl) is approximately 123.3 million tonnes annually (FAO, 2019), of which 8.1% is from Africa (FAO, 2019). The East African region produces approximately 1 million tonnes with a total area of around 0.1 million hectares under cultivation and the mean production of 11 tonnes per hectare (FAO, 2019). There are no clear production statistics of watermelon and pumpkin in Uganda but generally productivity of these crops is considered low (Kabunga et al., 2014).

The production challenges of horticultural crops in sub-Saharan Africa include the lack of improved varieties, pests, diseases, high cost of seeds, insufficient certified production inputs, lack of/limited proper storage facilities, price fluctuations, limited access to affordable sources of financing, lack of extension services, and poor crop management practices (Ddamulira et al., 2021; Waweru et al., 2019). The low production rates of watermelon and pumpkin in Uganda could be attributed to such biotic and abiotic constraints. Previously, pests and diseases were reported to be among the major constraints in the production of watermelon and pumpkins in Uganda (Masika et al., 2017). However, production constraints, management practices, sources of inputs, and the strategies implemented by farmers, are not clearly documented or unknown in Uganda.

Pumpkins and watermelons are widely cultivated for their edible leaves, fruits, and seeds, thereby contributing to food, nutrition, and income security in sub-Saharan Africa (Dinssa et al., 2016). Pumpkins are consumed in cooked form, while watermelons are consumed as ripe fruits. Both may also act as animal feed and as constituents of many commercial products because of their high nutraceutical values (Salehi et al., 2019). They are a good source of dietary fibre and have many healthy properties that help in reducing incidence of several morbidities due to high composition of unique phytochemicals (such as polyphenols and carotenoids) (Peiretti et al., 2017), proteins, carbohydrates, vitamins (such as K, B6, riboflavin, and thiamine), and oils (Kim et al., 2012; Peiretti et al., 2017). Pumpkins have a diverse variety of food uses depending on its stage of maturity (Bhat & Anju, 2013). Immature fruits and leaves are used as vegetables, while all other parts, such as seeds and mature fruits, have varied nutritional values and uses (Bhat & Anju, 2013). Further, dried products, pomades, pickles, and juices all containing high levels of essential amino acids make these cucurbits essential in addressing nutritional deficiencies (Elinge et al., 2012; Jacobo-Valenzuela et al., 2011; Rakcejeva et al., 2011; Vinayashree & Vasu, 2021).

The objective of this study was to assess the current production constraints for watermelon and pumpkin production in Uganda. Specifically, the study aimed at determining the major varieties grown, sources of seed, and the major constraints affecting production. This is important because of these crops’ high nutritional values, are attracting market regionally and in addition, pumpkin leaves are being used as vegetables. This research builds on a previous study that pointed to presence of viruses affecting production of watermelon and pumpkin in Uganda although other constraints were not documented. This will inform production of the crops were policy makers, farmers, researchers can base on to make decision for improved production of both watermelon and pumpkin and we point out research gaps that need attention.

## Methods

### Study areas

**Fig. 1:**
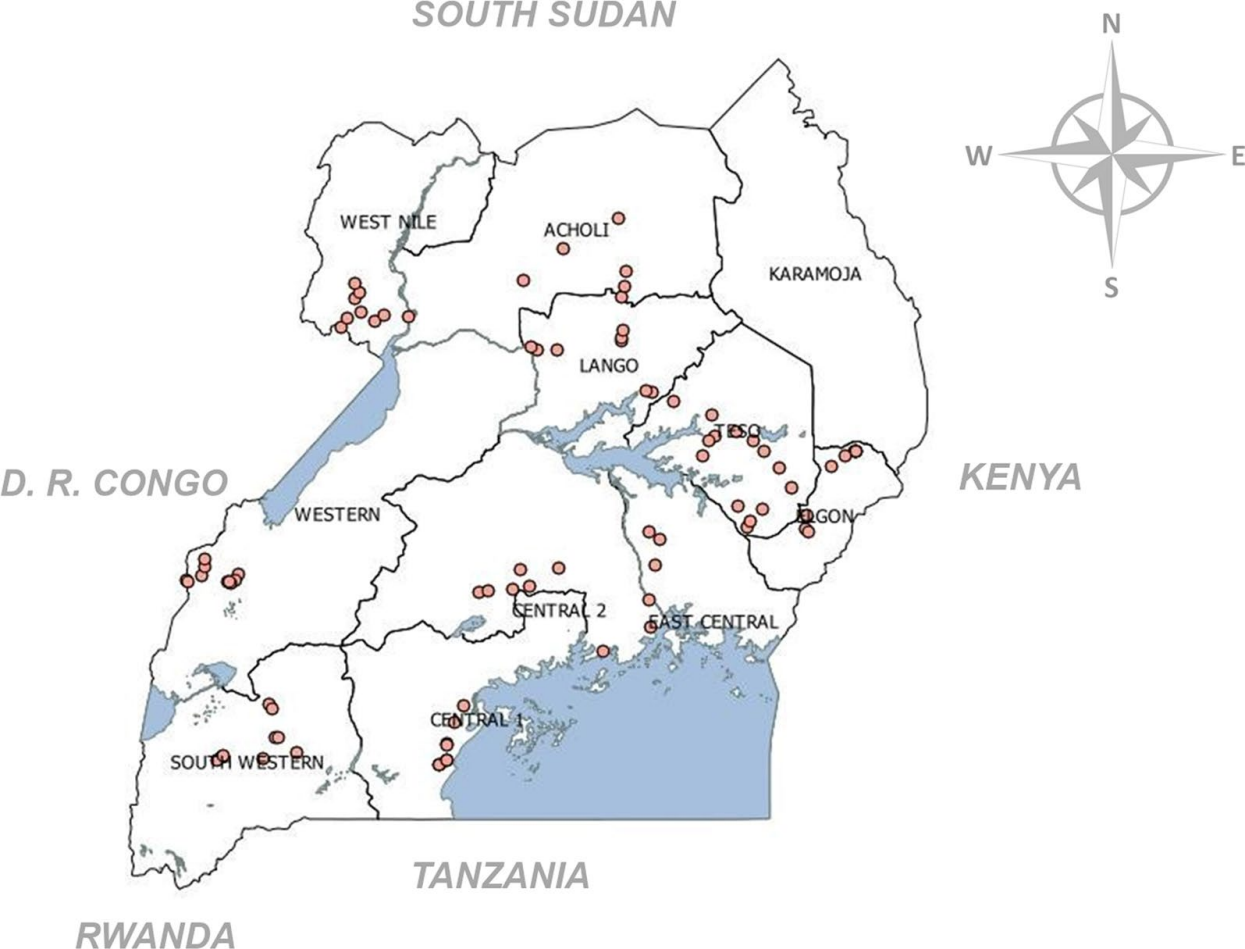
Map showing the study locations in Uganda (1.0667° N, 31.8833° E). The dots represent the location of fields where the observations were made and/or homesteads involved in the survey

Data were collected between July and November 2020 from 28 districts in nine of the 11 sub-regions of Uganda (Fig. 1, Table 1). The sub-regions (sampling units) were selected according to the importance of pumpkin and watermelon in their food production systems (Masika et al., 2017). The sub-regions (covering nine agro-ecological zones of West Nile, Mid North, South Eastern, Western Highlands, Southern Highlands, Southern drylands, Eastern, Lake Victoria crescent, and lake Albert crescent) differ from each other edaphically, farming systems, climatic factors, natural vegetation type, and altitude (Wortmann & Eledu, 1999). The main economic activity in the selected study area is agriculture, employing more than 70.0% of people (Mwesigye & Matsumoto, 2016; Odongo et al., 2017). The food crops grown in these areas include sweet potato (*Ipomoea batatas* (L.) lam), sorghum (*Sorghum bicolor* (L.) Moench, maize (*Zea mays* L., and bananas (*Musa* spp L.), and pumpkins as intercrops (Haggblade & Dewina, 2010; UBOS, 2009).

**Table 1:** The number of participant farmers sampled from different districts in the nine sub-regions in Uganda in 2020

Sub-region	District	Female	Male	Total
Southwestern	Ibanda	2	2	4
	Mbarara	1	2	4
	Bushenyi	2	2	3
Western	Bundibugyo	3	2	5
	Kabarole	4	0	5
	Kamwenge	4	0	3
West Nile	Arua,	2	1	3
	Nebbi	1	2	3
	Pakwach	2	1	3
Lango	Dokolo	1	2	3
	Lira	2	1	3
	Oyam	1	2	3
Acholi	Pader	2	1	3
	Gulu	2	1	3
	Kitgum*	–	–	
Teso	Serere	3	0	3
	Soroti	2	1	3
	Kumi	3	0	3
	Palisa	2	1	3
Elgon	Mbale	2	1	3
	Bulambuli	2	1	3
	Kween	3	4	7
Central	Mukono	5	1	6
	Masaka	8	3	11
	Mityana	3	0	3
	Luwero	3	0	3
East central	Jinja	2	1	3
	Kamuli	1	2	3
	Bugiri	1	2	3
Total		69	36	105

^*^Excluded from the survey because of the COVID-19 pandemic

### Sampling procedure

Areas where these crops are grown under open cultivation were identified from the administrative units of Uganda. Agricultural officers (AOs) at the sub counties of each selected district assisted with identification of 105 households in all the districts from the nine sub-regions. The first stage was purposeful selection of three districts in each sub-region. Three sub-counties were then chosen due to the importance and availability of watermelon and/or pumpkin fields. In each district, between three to eight fields were surveyed. The fields (measuring at least three quarters of an acre) were those of smallholder farmers who had cultivated either one or both of these crops for at least four consecutive seasons. This could be for two consecutive years or more as pumpkin and watermelon take from 80 to 120 days to mature and all the farmers in the surveyed regions can plant these crops twice a year. This facilitated easy articulation of constraints, management practices, sources of inputs, and the strategies to control them. The method of field selection used was the same as that used by Tugume et al. (2008) in the survey of wild plants of the family Convolvulaceae in Uganda. The distance between successive crop fields was determined according to the method used by Alicai et al. (2019) for survey of cassava brown streak disease caused by Cassava brown streak ipomoviruses in Uganda. This also ensured that the fields were not close to one another with an interval of 20–40 km between fields and therefore covering a wider area to clearly capture variations in disease incidence between locations (Scholthof et al., 2011). The plants in the surveyed fields were not less than one and a half months old for ease of identification of disease symptoms.

### Data collection

Data from the sub-regions were collected using a structured questionnaire. Varieties of pumpkin were identified using photographs of different varieties according to Nakazibwe et al. (2019). Information on the constraints, management practices, production inputs, and the strategies were catalogued, revised, pre-tested and uploaded to the mobile survey application KoBoCollect (Nampa et al., 2020; Palla et al., 2016) and installed on tablets and smartphones. Before the interview, the farmers was briefed on the objective of the study and consent was sought giving the farmer freedom to pull out of the interview any time. Field observations were made with the farmer on presence of different pests (aphids, beetles, melon fly, mites (*Tetranychus urticae,* C. L. Koch), rats (*Rattus rattus,* Linn), rindworms (*Spodoptera exigua,* Hubner) and *Trichoplusia ni* (Hubner), whitefly and cutworm) and disease (anthracnose (*Glomerella cingulata* (Stoneman) Spaulding & von Schrenk). downy mildew (*Peronospora sparsa* (Berkeley) Jaczewski), gummy stem blight (*Stagonosporopsis cucurbitacearum* (Fries) Aveskamp, Gruyter & Verkley), powdery mildew (*Leveillula cucurbitacearum,* Golovin), and virus diseases. We printed colour photographs of pests and diseases of watermelon and pumpkin and used them to help farmers in their identification.

### Data analysis

The data were exported and initially coded in Microsoft Excel V.2016, then imported into Stata v15.0 where all the analyses were performed. The proportion of males to females, pumpkin to watermelon farmers, farmers who planted one cucurbit crop to those who planted two, and those who practiced intercropping to those who did not, were analysed using t-test. The varieties grown by the farmers in the different sub-regions were analysed using one-way analysis of variance (ANOVA). The relationship between the source of seed, sale of produce, diseases, pests, and general constraints, control methods for pests and diseases were analysed using contingency chi-square tests with measures of association and the significance level inferred at 0.05.

## Results

### Varieties of pumpkin and watermelon grown in survey areas

**Fig. 2:**
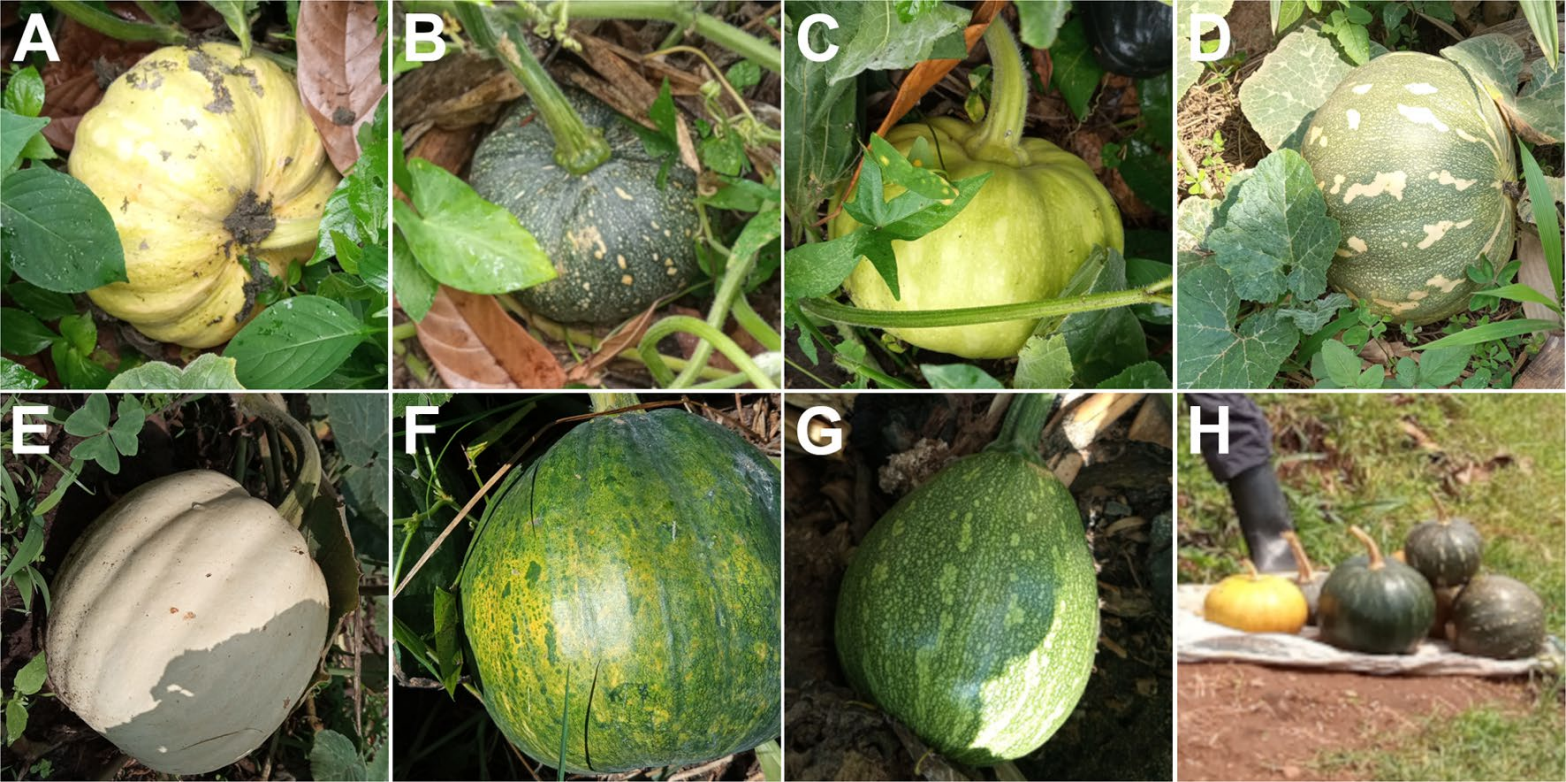
Fruit characteristics of some of the pumpkin varieties grown in the surveyed areas. **A** Wujju, **B** Dulu, **C** Ebihaaza, **D** Oziga, **E** Sweat cream, **F** Sweat pumpkin, **G** Sunfish, **H** Wujju, Dulu and Ozida on sale at a roadside stall

A total 11 varieties of pumpkin were recorded in the study. The most widely grown variety is “Dulu” (26.7%), followed by “Wujju” (24.4%), “Oziga” (16.7%), “Sweat cream” and “Sweety pumpkin” (7.8% each). Others grown by few farmers were “Larger white” and “Butternut” (4.4%, respectively), “sunfish” (3.3%), “Ebihaza” (2.2%), “Bala” and “Sugar pie” each 1.1%, respectively (Fig. 2). The differences in the mean number of farmers who planted these varieties in the study areas were statistically significant (P = 0.01; df = 8) (Table 2). “Dulu” variety was grown in all sub-regions except Teso while “Butternut” variety was grown only in West Nile sub-region. Most farmers of watermelon planted “Zebra” (86.7%), and the remaining (13.3%) planted “Chairman” variety (Table 2).

**Table 2:** Pumpkin varieties (Dulu 26.7%, Wujju 24.4% and Oziga 16.7% among others are most widely grown by farmers in the study areas

Pumpkin Variety	Regions									Total	Frequency	df	P
	Acholi	Central	East Central	Elgon	Lango	South western	Teso	West Nile	Western				
Dulu	1	3	5	4	1	2	0	3	5	24	26.7	8	0.01*
Large white	1	1	0	0	0	0	2	0	0	4	4.4		
Sweat cream	1	2	1	0	0	0	0	0	3	7	7.8		
Wujju	3	2	1	3	2	1	7	1	2	22	24.4		
Oziga	0	2	0	1	5	3	2	0	2	15	16.7		
Sweety pumpkin	0	2	1	1	1	1	0	0	1	7	7.8		
Sunfish	0	0	1	0	0	2	0	0	0	3	3.3		
Ebihaza	0	0	0	0	0	2	0	0	0	2	2.2		
Bala	0	0	0	0	0	0	1	0	0	1	1.1		
Butternut	0	0	0	0	0	0	0	4	0	4	4.4		
Sugar pie	0	0	0	0	0	0	0	1	0	1	1.1		

* Denotes significant difference

### Watermelon and pumpkin farming practices observed

A total of 105 farmers were surveyed from 28 districts in nine sub-regions during our study (Fig. 1; Table 2). The proportion of males involved in watermelon and pumpkin production in the study areas was 65.7% and their age bracket ranged from 25 to 75 years with a mean of 44.2 years. In contrast, the proportion of women was lower (34.3%) with an age range of 20 to 70 years and an average age of 46.1 years in all the sub-regions, with 44.8% being in the youthful stage of 40 years and below. The difference in the number of males to females involved in the cultivation of these crops was not statistically significant (P > 0.05). Furthermore, of the total number of farmers interviewed, 20.9% had no formal education compared to 63.8%, 13.3% and 1.9% that had attained primary, secondary, and post-secondary education, respectively. Watermelons was planted in Ngenge irrigation scheme, Kween district, from Elgon, and Masaka, Mityana and Mukono districts in the Central sub-region, while pumpkins were grown in all the sub-regions surveyed. The differences in the mean number of farmers of watermelon to those of pumpkin in the sub-regions were statistically significant (P < 0.001; t-test = 4.11) (Table 3).

**Table 3:** Predominant cropping systems of watermelon and pumpkin by gender in the study areas

Gender/ Production system	Regions									Total no. of farmers		%		Mean		SD		SE		t-test		CI	Probability
	Acholi	Central	East Central	Elgon	Lango	South Western	Teso	Western	West Nile														
Male	4	19	4	7	4	5	10	11	5	69		65.71		4.94		2.78		0.33		0.20		4.28 ± 5.61	0.84
Female	2	4	5	6	5	6	2	2	4	36		34.29		4.83		2.24		0.37		0.20		4.08 ± 5.59	0.84
Pumpkin only	6	12	9	9	9	11	12	13	9	90		85.71		5.3		2.57		0.27		4.11		4.76 ± 5.84	< 0.001*
Watermelon only	0	11	0	4	0	0	0	0	0	15		14.28		2.53		0.91		0.23		4.11		2.03 ± 3.05	< 0.001*
Intercropping ^a^	6	14	9	9	9	11	12	13	9	90		85.71		5.30		1.16		2.57		4.22		4.76 ± 5.83	< 0.001*

*SD* Standard deviation

*SE* Standard error

*CI* Lower and upper boundaries of confidence interval for the mean

^a^indicates intercropping with maize, sorghum, banana, cassava, yam, or tree crops

^*^denotes significant differences

**Fig. 3:**
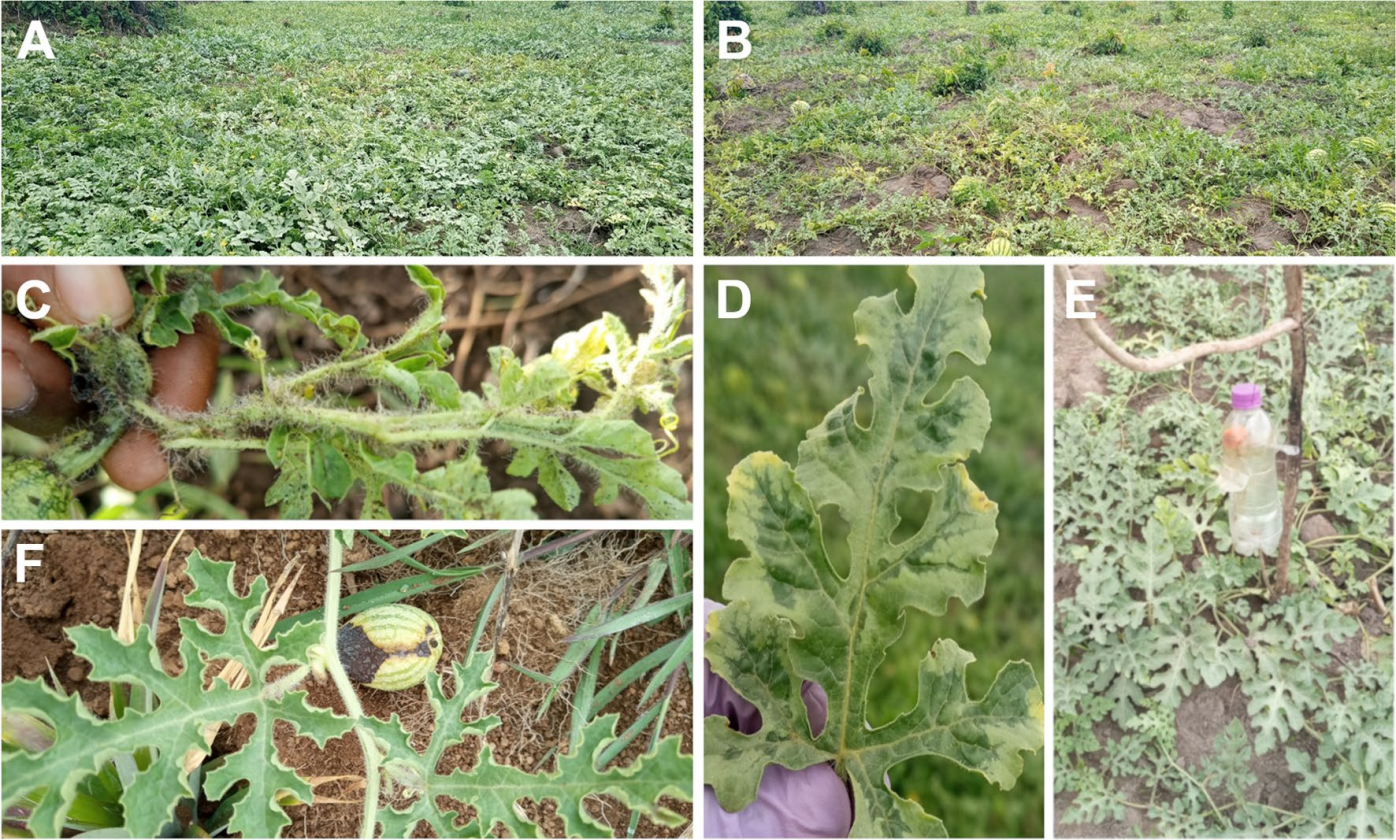
Watermelon cropping system of young (**A**) and aged (**B**) fields in Elgon sub-region; biotic production constraints and mitigation strategies recorded in this study. **C** Plant heavily infested by aphids  at Kizala Village in Mukono District, Central sub-region; **D** Virus-like disease symptoms on watermelon leaf at Kazimba Village in Masaka District, Central sub-region; **E** A local pheromone trapping of watermelon flies at Lukolo Village in Masaka District, Central sub-region; **F** Rotting disease at Nakoosi Village, Mukono District, Central sub-region

**Fig. 4:**
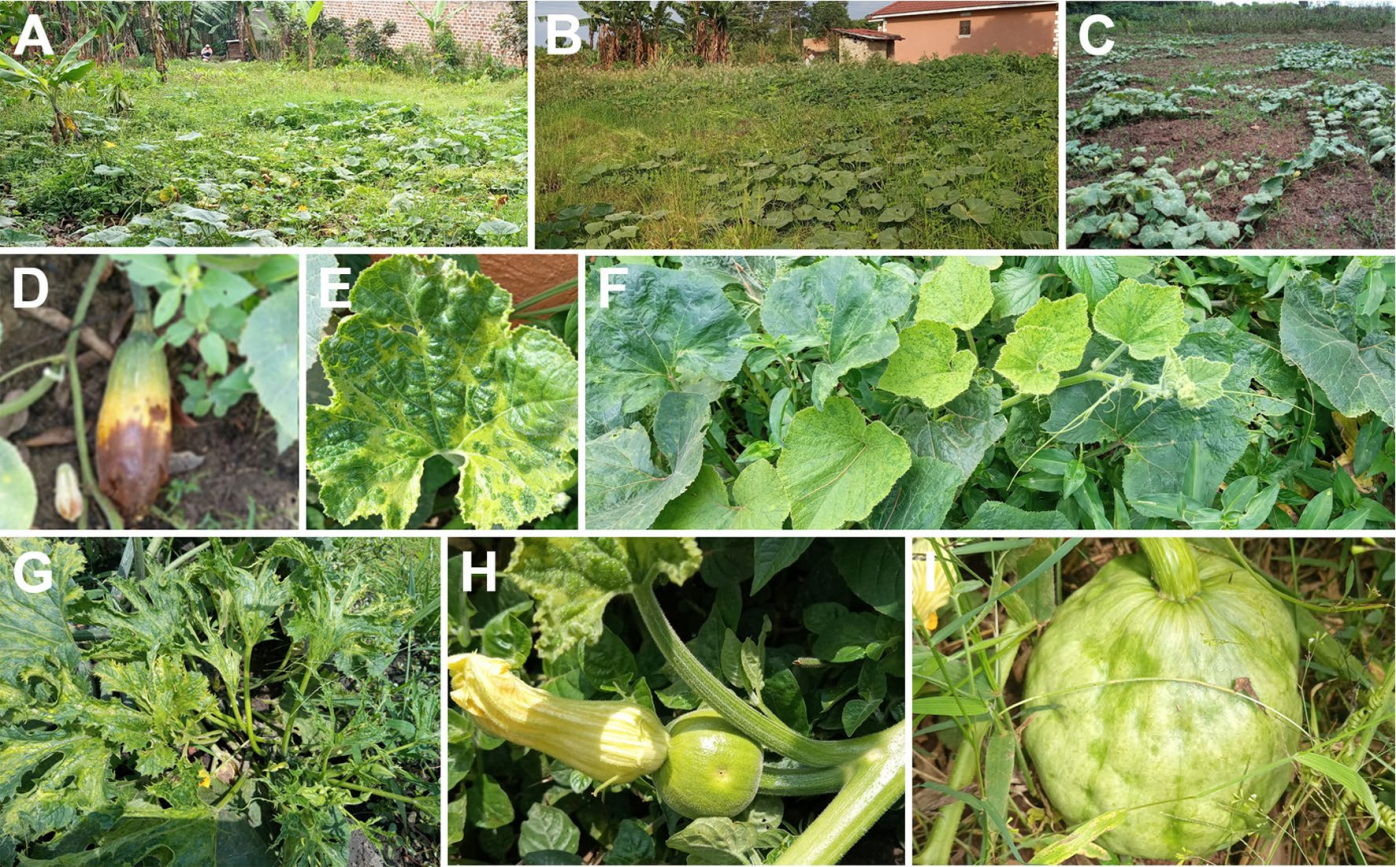
Pumpkin cropping systems, nutrient deficiency and virus-induced symptoms. **A** and **B** pumpkin in less managed backyard gardens next to homesteads often in banana plantations; **C** in rare cases, pumpkins may be planted in separate gardens next to annual crops; **D** fruit showing blossom end rot, characteristic of nutrient deficiencies, at Okolo Ayiju Village Arua district, West Nile sub-region; **E** Vein banding, yellowing, blistering and deformation in Okii-Oyere Village in Lira district, Lango sub-region and Apuche Achwa Village in Pader district, Acholi sub-region; **F** Chlorosis and leaf distortion in Masaka district and observed also in other sub-regions. **G** Rossete-like symptoms observed in Bulambuli district; **H** Young pumpkin fruit with pest-like symptoms at Kirundo Village in Bushenyi District, South Western sub-region. **I** A developing pumpkin fruit with a deformed rind jeopardising the quality, characteristic of virus-like infections, in Bugonzi Kabale in Masaka, Central sub-region

Furthermore, 81.0% of the farmers planted either pumpkin or watermelon only while a few farmers, (19.0%) planted both. The differences in the number of farmers who planted either of the crops in the study area was statistically significant (P < 0.001). Watermelon was mainly grown as a monoculture (13.3%) (Fig. 3), while pumpkin (85.7%) was mainly grown as an intercrop with other staple crops such as cassava (*Manihot esculenta,* Crantz), maize, banana, sweet potato, coffee (*Coffea arabica* L. & *C. canephora,* Pierre ex A. Froehner), and in some agroforestry systems (Fig. 4). The difference in the means of those who intercropped and those who did not were statistically significant (t-test = 4.22, P < 0.001) (Table 3). In addition, farmers planted pumpkin in the second season after harvesting crops like maize, rice, and millet (*Eleucine coracana*, Gaertn). Watermelon farmers planted the crop two times a year unless under crop rotation (data not shown).

### Source of seed for watermelon and pumpkin cultivation

It was observed that 69.5% of the pumpkin farmers used their own saved seeds as the source of planting materials compared to 14.3% of farmers who obtained seeds from agro-input shops. Several other farmers obtained seeds by buying (or as gifts) from fellow farmers and local markets. All farmers who planted watermelon in the Central and Elgon sub-regions indicated that they obtained seeds from agro-input shops which may not necessarily be certified. There was an association between the source of seeds and the sub-regions. These differences were statistically significant (χ = 68.54; df = 24; P < 0.001) (Table 4).

**Table 4:** Sources of seed and where farmers sell their watermelon and pumpkin fruits in surveyed areas in Uganda

Regions										Total	Frequency	df	χ^2^	P
	Acholi	Central	East Central	Elgon	Lango	South western	Teso	West Nile	Western					
Source of seed														
Agro-input shops	1	13	0	0	0	0	0	0	1	15	14.29	24	68.54	< 0.001*
Friend/fellow farmer	0	0	2	0	0	0	3	1	2	8	7.62			
Market	0	1	0	4	0	2	1	0	1	9	8.57			
Farm-saved seeds	5	9	7	9	9	9	8	8	9	73	69.52			
Market outlets														
Farm gate	0	14	0	2	0	0	0	0	0	16	15.24	16	59.59	< 0.001*
Local open market	5	3	6	9	3	8	8	7	8	57	54.29			
Traders	1	6	3	2	6	3	4	2	5	32	30.48			

*df* degree of freedom

χ^2^ chi-square value for the measure of association

*P* probability value

^*^denotes significant differences

Farmers sold their produce using different methods, for example, all the watermelon farmers and one pumpkin farmer sold the whole field (farm gate) (15.2%), while 54.3% sold their produce at nearby local markets and the others (30.5%) sold their produce to traders. The association of method of selling to sub-region was statistically significant (χ = 68.54, df = 24, P < 0.001) (Table 4).

### General constraints affecting watermelon and pumpkin production

The constraints affecting watermelon production in the surveyed areas were pests (66.7%), diseases (13.3%), floods (13.3%) and drought (6.7%). While pumpkin production in the surveyed areas was affected by diseases (52.2%), pests (31.1%), floods (3.3%), drought (4.8%) among others. Generally, the main constraints limiting both watermelon and pumpkin production in were pests (40.9%), diseases (34.3%), drought (8.6%), high transport costs (5.7%) and high labour costs (4.8%) respectively. Others are low yields (1.9%), price fluctuation (1.9%), limited market (0.9%) and poor soils (0.9%) (Table 5). In Acholi sub-region, diseases (Pader and Gulu districts) and pests (Pader district) are the most important constraints, while in central sub-region, pests (in all the districts), diseases (all districts), high labour costs (Mukono and Masaka), and drought (Mityana and Masaka) affect watermelon and pumpkin production. In East central, diseases (Kamuli and Jinja) are the most important constraints, while pests, high labour costs and diseases affect watermelon and pumpkin cultivation in Elgon sub-region. In the Lango sub-region, pests, transport, drought, diseases, price fluctuations, and poor soil respectively affect pumpkin production. Pests, diseases and high transport affect cultivation of pumpkin in South western sub-region, while pests, transport, diseases, drought, low yields are responsible for low productivity of pumpkin in the Teso sub-region. In west Nile, diseases, pests and drought as it is in Western subregion affect production of pumpkin. The differences in association of the production constraints with the different sub-regions were statistically significant (χ = 87.51, df = 64, P = 0.027) (Table 5).

**Table 5:** The number of respondent farmers who reported the overall production constraints (pests = 40.95%, diseases = 34.29% as most important), key diseases (bacterial wilt 21.9%, downy mildew 12.38%) and pests (whiteflies 29.50, aphids 20.00%) among others affecting watermelon and pumpkin production in the study areas in Uganda

Production constraints	Regions									Total	%	df	χ^2^	P
	Acholi	Central	East Central	Elgon	Lango	South western	Teso	West Nile	Western					
Overall														
Diseases	5	8	4	3	1	2	2	5	6	36	34.29	64	87.51	0.027*
Drought	0	1	1	1	2	0	1	1	2	9	8.57			
High labour costs	0	2	0	3	0	0	0	0	0	5	4.76			
High transport costs	0	0	1	0	2	1	2	0	0	6	5.71			
Low yields	0	0	1	0	0	0	1	0	0	2	1.90			
Limited market	0	0	1	0	0	0	0	0	0	1	0.95			
Pests	1	12	0	6	2	8	6	3	5	43	40.95			
Price fluctuation	0	0	1	0	1	0	0	0	0	2	1.90			
Poor soils	0	0	0	0	1	0	0	0	0	1	0.95			
Diseases														
Anthracnose	1	1	0	2	3	0	0	2	1	10	9.52	48	67.44	0.033*
Downy mildew	0	4	0	4	1	0	1	1	2	13	12.38			
Gummy stem blight	0	0	0	1	0	0	0	0	0	1	0.95			
Powdery mildew	0	4	2	0	0	2	0	0	0	8	7.62			
Virus-like disease	1	2	1	0	0	1	2	0	0	7	6.67			
Bacterial wilt	2	8	5	2	0	0	1	2	3	23	21.90			
Pests														
Aphids	1	2	3	0	4	4	3	2	2	21	20.00	64	153.3	< 0.001*
Beetles	0	1	1	4	0	0	0	0	0	6	5.71			
Melon flies	1	14	0	2	0	0	0	0	0	17	16.19			
Mites	0	2	0	0	0	0	0	0	0	2	1.90			
Rats	0	2	2	0	0	0	0	0	4	8	7.62			
Rindworm	1	0	0	0	0	0	0	0	0	1	0.95			
Whiteflies	2	1	3	4	3	5	7	3	3	31	29.52			
Cutworm	1	0	0	3	2	2	2	0	0	10	9.52			
Red ants	0	1	0	0	0	0	0	4	4	9	8.57			

*df* degree of freedom

χ^2^ chi-square value for the measure of association

*P* probability value

^*^denotes significant differences

### Disease affecting watermelon and pumpkin

The diseases that affect watermelon in the surveyed areas included bacterial wilt (*Erwinia tracheiphila,* Smith) (33.3%), downy mildew (20.0%) viruses-like diseases (13.3%), compared to the diseases that affected pumpkin production as bacterial wilt (21.1%), downy mildew and anthracnose (each 12.2%), powdery mildew (7.8%), viral diseases (5.6%). A large proportion of respondent farmers (20.0% for watermelon and 41.1% for pumpkin) could not specify the diseases affecting their crops. In general, bacterial wilt (21.9%), downy mildews (12.4%), anthracnose (9.5%), powdery mildews (7.6%), virus diseases (7.6%), and gummy stem blight (1%) (Fig. 4) affect watermelon and pumpkin productivity in the surveyed areas. In Acholi sub-region, bacterial wilt (Pader and Gulu districts), virus diseases (Gulu), and anthracnose (Pader) respectively affect pumpkin. However, in Central sub-region, bacterial wilt (Masaka, Mukono, Luwero, and Mityana) powdery and downy mildews (Luwero, Masaka and Mukono), virus diseases (Masaka), and anthracnose (Masaka) affect production of both pumpkin and watermelon. In East central, bacterial wilt was the most common disease affecting pumpkin production in Kamuli and Bugiri and powdery mildew in Jinja. However, in Elgon sub-region, downy mildew an important disease affecting both watermelon and pumpkin production was reported in Mbale, Bulambuli and Kween. Other diseases in this sub-region included anthracnose and bacterial wilt. In Lango sub-region, anthracnose was recorded in Dokolo and Oyam district and downy mildew in Lira district. Powdery mildews and virus diseases affects pumpkin production in South western sub-region. Virus diseases, anthracnose, and bacterial wilt affect production of pumpkins in Teso sub-region while bacterial wilt, downy mildew and anthracnose are responsible for low production of pumpkin in West Nile similar to the Western sub-region (Table 5). The diseases where highly associated with some sub-regions and these were statistically significant (χ = 67.44, df = 48, P = 0.033) (Table 5).

### Pests affecting watermelon and pumpkin production

There was a variation in incidence of pests affecting watermelon and pumpkin in the surveyed areas. In watermelon, key pests included melon flies (63.2%), rats and cut worms (10.5% each), and whiteflies and beetles (5.2% each). In pumpkin fields, whiteflies (35.6%), aphids (22.2%), rats (6.7%) were the most important pest constraints. However, pests affecting both pumpkin and watermelon included whiteflies (29.5%), aphids (20.0%), melon flies (16.2%), and cutworms (9.5%) (Table 5). In Acholi sub-region, aphids, melon fly, rindworms and cutworms affected pumpkin production in Pader and Gulu respectively. However, melon flies, rats, affected watermelon production in Masaka, Luwero and Mukono districts while the pests observed on pumpkin included aphids, beetles, melon flies, mites and white flies. Similar results were observed from the Elgon sub-region including melon flies, cutworms and beetles. However, for pumpkin, the key pests in Elgon sub-region were beetles, whiteflies and rats. In East central sub-region, there was no clear association of any pest to a district. Aphids, beetles, rats and whiteflies affected pumpkin productivity. Aphids and whiteflies where uniformly observed in the fields surveyed in Dokolo, Lira and Oyam districts of Lango sub-region. In South western, Teso, West Nile and western sub-regions, the key pests were whiteflies, aphids, cutworms, red ants, and rats. The differences in the associations of the pests with the sub-regions were statistically significant (χ = 153.3; df = 64; P < 0.001) (Table 5).

### Management of biotic constraints

Weeding using a hand hoe (74.2%) was the most commonly reported method used by farmers to manage weeds in all the sub-regions. Others used herbicides (5.7%) while others did not apply any method to manage weeds (1.9%) most especially in East central sub-region. Further, one farmer used a combination of weeding and herbicides in the Central sub-region. The different weed management strategies were more associated with some sub-regions and not others (χ = 39.65; df = 32; p = 0.0078 (Table 6). The main control method for diseases was the use of chemicals (19.1%). All farmers who planted watermelon used fungicides, bactericides (like cuprous oxide (Cu_2_O and cuprous oxide/zinc oxide (Cu_2_O/ZnO) (*data not shown*). However, most pumpkin farmers did not apply chemical sprays for disease control (Table 6). To control pests, 63.2% of the farmers used ash (organic powder obtained after burning plant remains) across all the sub-regions surveyed. Other methods included the use of pesticides (7.5%), pheromones (17.1) (Table 7). Some pumpkin farmers (12.4%) did not apply any pest management option. Pheromone traps (16.0%) were used to trap melon flies by watermelon farmers. Others are cultural control measures like clearing the borders of their farms. The different methods of pest management were associated with specific sub-regions. These differences were statistically significant (χ = 46.6; df = 4; P < 0.001) (Table 6).

**Table 6:** Control measures used by farmers in the management of diseases, weeds, and pests affecting watermelon and pumpkin crops in the study areas

Control measure	Regions									Total	%	df	χ^2^	P
	Acholi	Central	East Central	Elgon	Lango	South Western	Teso	West Nile	Western					
Weeds														
None	0	1	0	0	0	1	0	0	0	2	1.90	32	39.65	0.0078*
Weeding	4	12	7	9	8	8	11	7	13	79	74.2			
Herbicides	2	0	1	0	2	1	0	0	0	6	5.7			
Weeding and herbicides	2	10	1	4	0	1	1	1	0	18	17.1			
Pests														
None	2	6	0	0	0	2	2	2	0	14	13.2	4	46.6	< 0.001*
Ash	3	1	6	10	8	8	10	8	13	67	63.2			
Pesticide	0	5	0	3	0	0	0	0	0	8	7.5			
Pheromones	0	15	0	2	0	0	0	0	0	17	16.0			
Disease														
None	5	12	8	9	8	10	11	7	13	83	79.0	8	10.96	< 0.001
Chemical sprays	1	11	1	4	1	1	1	2	0	22	21.0			
Method of chemical application														
Mixing of herbicides and pesticide	1	10	2	3	1	1	1	1	0	20	19.1	8	14.67	0.0696
No mixing of pesticides and herbicides	5	13	7	10	8	10	11	8	13	85	80.9			

*df* degree of freedom

χ^2^ chi-square value for the measure of association

*P* probability value

^*^denotes significant differences

**Table 7:** Strategies for the control of disease, pests, and weeds in watermelon and pumpkin production in Uganda

Strategy	Regions									Total	Percentage	df	χ^2^	P
	Acholi	Central	East Central	Elgon	Lango	South western	Teso	West Nile	Western					
Weeding	1	2	0	5	7	11	11	5	7	49	46.67	24	159.47	< 0.001*
Weeding, ash	3	1	0	0	0	0	0	3	6	13	12.38			
Weeding, crop protection chemical sprays	2	17	0	8	2	0	1	1	0	31	29.52			
Weeding, crop rotation	0	3	9	0	0	0	0	0	0	12	11.43			

*df* degree of freedom

χ^2^ chi-square value for the measure of association

*P* probability value

^*^denotes significant differences

### Use of agrochemicals in the management of biotic constraints

Farmers reported having limited or no knowledge of agro-chemicals they were applying on their crops. Most farmers could not differentiate between herbicides, pesticides, and fungicides used against diseases, pests, weeds or as nutrient boosters. The mixing of different agro-chemicals was reported during the survey by 19.1% of the farmers most of whom had planted watermelon. The other farmers (80.9%) did not apply chemicals to manage biotic constraints. The differences in the way farmers managed biotic constraints were not statistically significant (Table 6).

When asked about pest, weed and disease control for next season, most farmers (46.7%) in eight of the nine sub-regions reported that they intend to use weeding alone to control weeds. In addition to this was the use of weeding and chemical sprays, which included herbicides, pesticides, and some fungicides (29.5%) in six sub-regions. Other strategies were the use of weeding and ash (12.4%), in four sub-regions, and the use of weeding and crop rotation in two sub-regions (11.4%). The associations of the different strategies with some sub-regions were statistically significant (χ = 159.5; df = 24; P < 0.001) (Table 7).

## Discussion

In this study we documented the varieties of watermelon and pumpkin grown by the farmers, farming practices, sources of seed, diseases, pests, general constraints, control methods for pests and diseases affecting their production in Uganda. From the study, 45.7% of the farmers were in the youthful stage (between 20 to 40 years). The highest proportion of farmers were males involved in watermelon and pumpkin production, similar to what was reported in Kenya (Isaboke et al., 2012). However, women were the highest gender found tending the crops and they play an important role in smallholder agriculture (Garwe et al., 2009). Furthermore, the involvement of youths in agriculture albeit its low levels is a positive feature and if the constraints are addressed, it can be a source of employment and boost the agricultural sector as also observed by Adekunle et al., (2009) in Kware state Nigeria.

The pumpkin varieties grown were local varieties with names of varieties based on the geographical distribution and diversity of local dialects and or languages (Missihoun et al. 2012) used by the farmers in management and selection of genetic resources. Farmers could select mature fruits of the variety of interest and subsequently select seeds for planting. The preference for some varieties over others was based on the dry matter content as reported by Priori et al. (2018), fruit texture leaf size and yield (Mbogne et al., 2015). Hybrid varieties of watermelon including “zebra” and “chairman” were grown in the surveyed areas in Kween and Central sub-region which were preferred for their sugar content, size and available market. According to this study, 85.7% of farmers intercropped pumpkin with other crops including cassava, maize, banana, sweet potato, coffee and some agroforestry systems as previously observed in Uganda (Masika et al., 2017). Intercropping of pumpkins with other crops is preferred to avoid high labour requirements for tending the pumpkin alone and also need to maximise output from a small area under production. Similarly, Ezin et al. (2021) reported that 85% of farmers intercropped pumpkin with other crops in Benin. Mixed cropping is believed to reduce pest build up by breaking their cycles through reducing the attractiveness of the general environment (Fanadzo et al., 2018). In contrast, some crops in the mixed farming system may shade infected plant residues that transmit diseases to other susceptible plants, e.g., pollen transmitted tobacco streak virus disease caused by *Tobacco streak virus* (Bhat and Rao 2020). Also, mixed farming allows alternative hosts for pathogens in close proximity causing diseases of crops they are intercropped with. Monocropping in watermelon was preferred because it was believed to increase planting density, reduce competition for nutrients, light, water, which in turn may lead to high production (Gebru, 2015). However, continuous monocropping may lead to changes in soil microbiota, chemical and enzymatic properties which may result into increase in harmful microbes and plant autotoxins hence incidence of soil borne diseases (Chen et al., 2020; Li et al., 2019).

Most pumpkin farmers used their own saved seeds and they recycle seasonally. Such seeds always contain admixtures are not always true to type, they may contain seeds of other plants and other substances which may affect germination rates over time, resulting into low plant vigour and production as reported in Ghana by Osei et al., (2020). Furthermore, sharing of seed by farmers encourages gene flow and hence survival of the species and maintenance of genetic diversity. In contrast, watermelon farmers obtained seeds from agro-input shops which may not be free from pathogens because they are mostly not certified. This calls for establishment of clear seed testing, monitoring and certification systems in Uganda to ensure authentic seeds on market (Reinker & Gralla, 2018). The use of clean and resistant planting materials should be encouraged to improve production reduce insect vectored diseases (Janse & Wenneker, 2002).

Across the nine sub-regions studied, pests and diseases were the most important constraints affecting cucurbit production, similar to the reports on these crops in other countries e.g., Japan and India (Davis et al., 2008; Reddy & Zehr, 2004; Rubatzky & Yamaguchi, 2012; Singh et al., 2017). Fungal, bacterial, and viral diseases were the most commonly reported diseases affecting cucurbits, as has also been reported in other sub-Saharan African countries like Sudan, Kenya, and Tanzania (Desbiez et al., 2016; Gorter, 1966; Kidanemariam et al., 2019; Lecoq et al., 2016; Mohamed et al., 1995). Plant virus diseases reduce optimal plant growth, yields, fruit quality, reproduction and susceptibility of the watermelon and/ or pumpkin to other pathogens which result in significant economic losses (Lecoq & Katis, 2014). The increase in transboundary trade of watermelon and pumpkin fruits which was recorded, intensive production systems, coupled with global warming may lead to further incidence of virus diseases (Jones, 2021). The inability of farmers to identify virus-like disease symptoms on their own may also imply inability to manage these diseases as was previously reported (Ibaba et al. 2015). Bacterial diseases on the other had cause enormous loses to pumpkin and watermelon and up to 80% losses have been recorded in cucurbits in the United States of America (Rojas et al., 2015). Symptoms of pests and diseases identified in our study calls for concerted efforts in their management if the watermelon/ pumpkin industry is to be economically viable. Therefore, there is need to strengthen local extension services and encourage formation of farmer groups to improve knowledge on pests and disease symptom identification and market performance (Ochieng et al., 2018). Farmers should also be encouraged to keep their fields clean by removing of volunteer and old plants, to reduce re-infection from such sources (Jones, 2006; Sastry & Zitter, 2014).

Generally, insects account for 15–25% yield losses in crops (Rathee & Dalal, 2018). Some insects are important vectors for many of the bacterial and viral diseases affecting plants (Dietzgen et al., 2016; Nagaraju et al., 2002; Buteme et al., 2020) while others feed on the plants directly or both. The most widely reported insect pest in watermelon fields in Uganda was the melon fly, which affects over 125 species of plants mainly in the family cucurbitacea. The female oviposits over 1000 eggs in the young soft and tender fruits which hatch into maggots that develop inside the fruit. The stinging provides a source of entry for fungal and bacterial pathogens causing devastation of up to 100% in cucurbit fruits (Dhillon et al., 2005). Evidently from this study, farmers had limited knowledge of symptoms caused by the insect vectors nor the association between the insect vectors and disease incidence. The most widely identified insect vectors, mainly by watermelon farmers, were whiteflies, followed by aphids, and melon flies. Pesticides and pheromone traps were mainly used by watermelon farmers, while most pumpkin farmers used ash as a method of pest management, similar to what was reported by Dhillon et al. (2005) and Huis, (2009). Notably, one drawback reported on the use of pheromones was that they were not able to trap all the adult insects which they attracted, while in other cases, the flies could potentially destroy the fruits before being trapped. A similar observation was made by Sarwar (2014) on the study of insect pests of summer vegetables, their identification, occurrence, damage, and the adoption of management practices in Pakistan. Most watermelon farmers mixed agrochemicals to spray their crops. This poses health concerns because of the chemical residues on the fruits and non-target borders or intercrops, as has been reported in tomato and other fruits in Uganda and other countries (Essumang et al., 2013; Kaye et al., 2015; Pedlowski et al., 2012; Ternest et al., 2020). This concern was emphasized further by the fact that many farmers using the agrochemicals (e.g., pesticides, herbicides, fungicides) were unable to differentiate between the different agrochemicals and their intended use, but instead used them indiscriminately in pest management. Therefore, farmers should be trained on the use of pesticides, herbicides, fertilisers, and fungicides, to minimize inappropriate application of these agrochemicals and associated negative health and/ or environmental effects (De Bon et al., 2014; Pretty & Bharucha, 2015; Tripathi et al., 2020). Indeed, in Uganda, application of agrochemicals in watermelon fields close to water bodies easily results in the deterioration of water and environment as well as a loss of the fauna that inhabits these lowlands (Amulen et al., 2017; Lema et al., 2014; Nakangu & Bagyenda, 2013). Also, since the highest percentage of farmers had attained primary education, they had limited knowledge in pest and disease management for their crops. Therefore, using agro-chemicals could proof difficult since it requires reading, comprehension and interpretation of instructions on the chemical to be used as also found in Ghana (Oduro-Ofori et al., 2014a, b). Watermelon was mainly planted in the low-lying areas close to Lake Victoria in the central sub-region or close to water bodies such as the Ngenge River in the Elgon sub-region. It was believed that planting watermelon in these low-lying areas provides sufficient moisture and water for irrigation during dry conditions (Turyahabwe et al., 2013).

The farmgate method of selling watermelon reported during the study was a preferred approach because it saved the farmer’s time of selling and processing the products for sell so that they concentrate on what they know best which is planting and tending the crops. This is similar to the finding by Gale (1997) while looking at direct farm marketing as a rural tool in the USA. Farm gate selling eliminates high transit losses due to bruising, cracking, and rotting. This is extubated by some poor roads for example the Mbale to Moroto road which farmers in Ngenge irrigation scheme (https://www.mwe.go.ug/library/supply-and-installation-5no-metrological-stations-5-irrigation-schemes-wadelai-pakwach) use to transport their produce (Yakubu et al., 2018). Farmers sold their fruits to traders who transported the fruits to nearby larger trading centres or exported the fruits to neighbouring countries (Kenya and South Sudan). This implies that they have the potential to generate income in the form of exports when production is improved and constraints addressed. Farmers also highlighted the high prices of inputs, lack of storage facilities, and lack of value addition to watermelons as some of the constraints. It is noteworthy that pumpkin farmers in West Nile also sell pumpkin leaves as vegetables in nearby markets, similar to what has been reported in other African countries like South Africa, Congo (Musotsi et al., 2003; Oboh & Aigbe, 2011). In addition, it was observed that some farmers kept their pumpkin fruits for some time after harvest, similar to what has been reported previously that pumpkins can be stored for more than three months without losing much of their nutritional value (Provesi et al., 2011).

Generally, farmers reported losses in the sale of their produce due to limited market, partly blamed on the COVID-19 pandemic travel restrictions. The use of hand hoe for weed management as a strategy in the coming season is a common indigenous knowledge practise in most sub-Saharan African countries (Ajani et al., 2013). This is partly because agro-chemicals are expensive and are used only on cash crops according to the farmers hence, much as pumpkin is slowly gaining market in urban areas, it is not considered a cash crop like watermelon (Huis, 2009). Watermelon farmers considered chemical based method of weed, pest, and disease control to be more effective and faster compared to alternative methods of control. When the constraints mentioned above are addressed, watermelon and pumpkin are potential food, income and nutrition security crops.

## Conclusions

This study highlights the importance of watermelon and pumpkin as sources of food, income, and nutrition security for local communities, even when these are not priority crop commodities in Uganda. The predominant variety of pumpkin is “Dulu”, while others were “Wujju” and “Oziga” out of the 11 varieties identified during the study. For watermelon, two varieties, “Zebra” and “Chairman” were grown by the farmers in the areas surveyed. Pumpkin farmers mainly used own saved seed, while those of watermelon obtained seed from agro-input shops and still others bought from neighbours, or received as gifts. There was low production of pumpkin which may be partly due to poor quality farm saved seed affecting profitability. The use a hand hoe to remove weeds was the main method of weed control with a few farmers using herbicides while farmers mainly used ash as the main method of pest control. The farmers of watermelon practised farmgate method of selling their produce while those of pumpkins sold to nearby markets, and local traders.

Many farmers were unable to distinguish between diseases affecting watermelon, pumpkin and their associated symptoms. Therefore, there is a need to train farmers on integrated pest and disease management, as well as in the appropriate methods for applying agrochemicals. Further, the development of local training materials (pest and disease symptom identification kits) that can be used to train farmers is paramount. Several interventions should be implemented to boost production of these crops so that the values of the crops are realised even more; for example, establishing storage centres for watermelon in the newly established irrigation schemes (e.g., Ngenge, Tochi, Doho, Mubuku, and Wadelai irrigation) and in Central Uganda where high production is expected. Other interventions include establishing certified seed systems which provide disease-free planting materials, addressing the issues of transport, and value addition. Lastly, identifying the causal pathogens of the diseases should be carried out so that management strategies are developed. For example, developing and/ or promoting a more environmentally friendly method of breeding for resistance to pests and diseases is paramount if improved production is to be realized.

## Data Availability

The data presented in this study are available within the article and additional materials.
